# Utility of Ultrasonography in Assessing the Effectiveness of Extracorporeal Shock Wave Therapy in Insertional Achilles Tendinopathy

**DOI:** 10.1155/2016/2580969

**Published:** 2016-11-28

**Authors:** Yi Cheng, Jian Zhang, Yehua Cai

**Affiliations:** ^1^Department of Ultrasound in Medicine, Huashan Hospital Affiliated to Fudan University, Shanghai, China; ^2^Department of Sports Medicine, Huashan Hospital Affiliated to Fudan University, Shanghai, China

## Abstract

*Introduction*. The aim of this study was to investigate the utility of ultrasonography (US) for predicting and assessing the effectiveness of extracorporeal shock wave therapy (ESWT) in insertional Achilles tendinopathy (IAT).* Methods*. A total of 42 patients with an established diagnosis of chronic IAT were examined by US before ESWT and at 4 weeks and 12 weeks after ESWT. The thickness and cross-sectional area (CSA) of the Achilles tendon, size of calcific plaques, tendon structure score, and neovascularization score were measured at each time point.* Results*. After therapy, Victorian Institute of Sport Assessment-Achilles (VISA-A) scores increased significantly, and the size of calcific plaques decreased (*P* < 0.05). Neovascularization scores increased at the 4th week and then decreased at the 12th week (*P* < 0.05). The thickness, CSA, and structure of the Achilles tendon did not change. Variables observed by US at baseline were not associated with changes in VISA-A scores at follow-up. However, the changes in calcific plaque size and neovascularization scores were related to the improvement of VISA-A scores between pre- and posttherapy (*P* < 0.01).* Conclusion*. Ultrasonography can reveal some changes in the insertion of the Achilles tendon after ESWT, but the outcome of ESWT in IAT cannot be predicted by the variables observed by US.

## 1. Introduction

Achilles tendinopathy (AT) is a frequent overuse problem in athletes and is also common in the general population [1]. From a functional perspective, it is helpful to classify AT occurring at the bone-tendon junction as insertional and those that occur more proximally as noninsertional. Insertional Achilles tendinopathy (IAT) is clinically characterized by tendon pain and swelling in the posterior heel with impaired performance [2].

Insertional Achilles tendinopathy is often difficult to treat, and there is no agreement on the best form of management. Recently, extracorporeal shock wave therapy (ESWT) has been proposed as an effective intervention and should be considered for IAT when other nonoperative treatments have failed [2–6]. The effectiveness of ESWT is assessed by questionnaires, such as the Victorian Institute of Sport Assessment-Achilles (VISA-A) questionnaire and the visual analog score (VAS) [3, 4, 6].

As a convenient, safe, and inexpensive examination, ultrasonography (US) has been widely used in the diagnosis of Achilles tendinopathy [7–11]. However, whether US imaging can reveal changes in the Achilles tendon after ESWT or predict or measure the outcome of ESWT remains unclear. The purpose of this study was to investigate the utility of US for predicting and assessing the effectiveness of ESWT in IAT.

## 2. Methods

### 2.1. Patients

Patients with an established diagnosis of chronic IAT who were eligible for ESWT were recruited from the sports medicine department of a large teaching hospital. The diagnosis of IAT was made clinically by a sports medicine doctor. For this study, IAT was defined as symptoms of moderate to severe posterior heel pain located at the bone-tendon junction that extended no more than 2 cm proximal from the base of the heel, swelling, and impaired function [2]. All patients had an established diagnosis of IAT for at least 6 months and had failure with at least 3 forms of traditional nonoperative treatments (such as rest, nonsteroidal anti-inflammatory drugs, physical therapy, and injections) for a minimum of 6 months. Patients were excluded if they had systemic illness or any other condition that could contribute to posterior ankle pain, such as ankle arthritis or radiculopathy. Patients with deformities of the ankle or a history of previous Achilles tendon rupture or surgery were also excluded. The study was approved by the local medical ethics committee, and informed consent was obtained from each subject prior to the investigation.

### 2.2. ESWT

All patients received ESWT with a radial shock wave device (EMS Swiss Dolor-Clast, Munich, Germany). In our study, ESWT was performed once a week for five weeks. At each of the five sessions, 2000 impulses (0.16 mJ/mm^2^) at 6–8 Hz were applied. With this device, starting at the area of maximal tenderness, shock waves were focused on the bone-tendon insertion and extended in a circumferential pattern.

### 2.3. Ultrasonographic Examination

**Figure 1:**
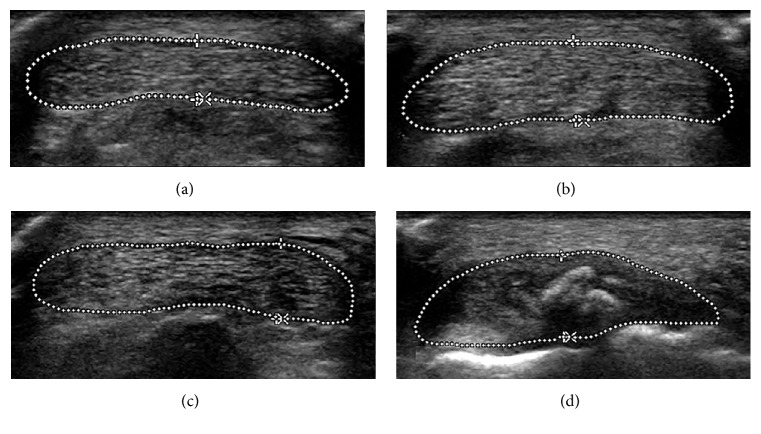
Transverse sonograms show 4-grade scale of tendon structure (outline of Achilles tendon traced by dotted line). (a) Grade 0—normal structure (homogenous echogenicity). (b) Grade 1—light structural changes (discrete hypoechogenic areas). (c) Grade 2—moderate structural changes (some well-defined hypoechogenic areas). (d) Grade 3—severe structural changes (extended hypoechogenic areas).

Ultrasonography scanning was performed with an iU22 ultrasonography unit (Philips Medical Systems, Bothell, Washington) by a radiologist who was blinded to the clinical status of the patients. A wide-frequency linear array transducer (5–17 MHz) was used. The patients were examined in a prone position with the foot hanging over the edge of the examination couch. All Achilles tendons were scanned in the transverse and longitudinal planes within 2 cm above the tendon insertion. Particular attention was paid to maintaining the ultrasound beam perpendicular and minimal probe pressure. The anteroposterior thickness and cross-sectional area (CSA) of the Achilles tendon were measured in the transverse plane 1 cm above the lowest rim of bone-tendon junction. We used a 4-grade scale to evaluate the tendon structure: 0—normal structure (homogenous echogenicity), 1—light structural changes (discrete hypoechogenic areas), 2—moderate structural changes (some well-defined hypoechogenic areas), and 3—severe structural changes (extended hypoechogenic areas) (Figure 1) [12]. A standardized, preprogrammed scanning protocol was used to obtain low blood flow. The color gain was adjusted to the maximum level, creating no clutter or noisy artifacts, and the pulse repetition frequency was set at low. Neovascularization was assessed based on the appearance of vessels inside the tendons: 0—no neovascularization, 1—1 vessel mostly in the anterior part, 2—2 vessels throughout the tendon, 3—3 vessels throughout the tendon, and 4—>3 vessels throughout the tendon [13]. If there was calcific plaque within 2 cm above the tendon insertion, the maximal diameter of the calcific plaque was measured.

Before the first session of ESWT, the baseline measurement was established. Follow-up examinations were performed at 4 weeks and 12 weeks after the whole sessions.

### 2.4. VISA-A Score

Every patient completed the VISA-A score at baseline and 4 weeks and 12 weeks after the treatment. The VISA-A questionnaire is valid and reliable to measure the severity of Achilles tendinopathy. The VISA-A score contains eight questions that cover the three domains of pain, function, and activity (a maximum score of 100) [14].

### 2.5. Statistical Analysis

Statistical analysis was performed using the SPSS 16.0 software program. A paired Student *t*-test or the Wilcoxon signed-rank test was used to compare the difference between baseline and posttreatment effects. Spearman's correlation was used to assess the relationship between outcome variables. Statistical significance was specified as *P* less than 0.05.

## 3. Result

Forty-two consecutive patients (29 males and 13 females) with a mean age of 37 years (range, 22–66 years) were enrolled in this study. The average duration of the disease was 10.2 ± 8.7 months (range, 6–37 months). Before ESWT, the mean VISA-A score was 54.0 ± 8.0 (range, 42–67). In the sonographic images, a hypoechoic degenerative region within the thickened insertion of the Achilles tendon was documented in all patients; in addition, 52.4% of patients had calcific plaques, and 73.8% of patients had neovascularization at the end of the Achilles tendon.

**Figure 2:**
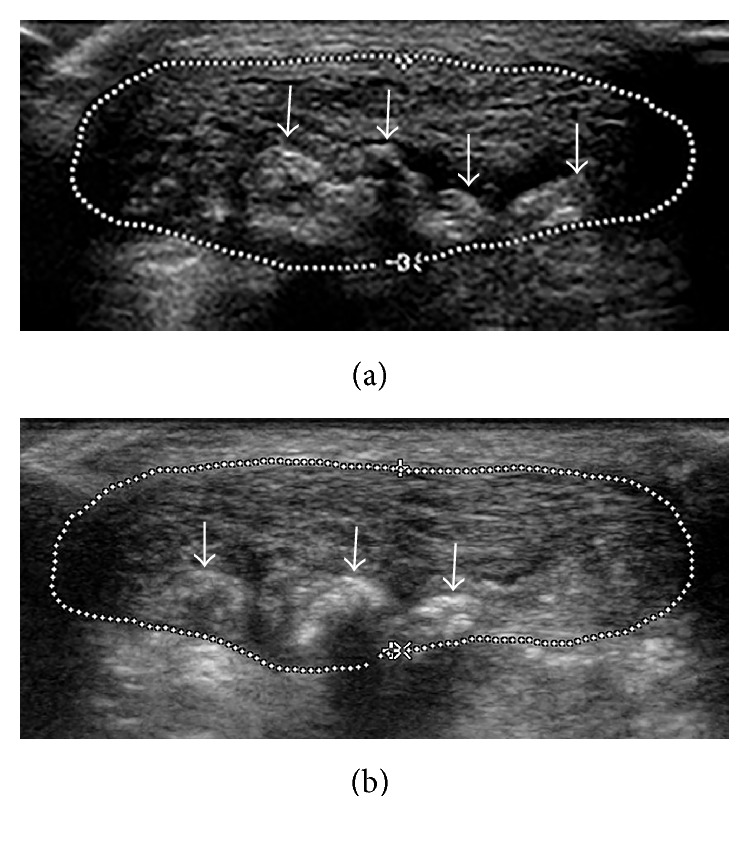
(a) Transverse sonogram shows plentiful calcific plaques (arrows) in the insertion of the Achilles tendon before therapy (outline of Achilles tendon traced by dotted line). (b) The calcific plaques reduced by the 4th week after therapy.

**Figure 3:**
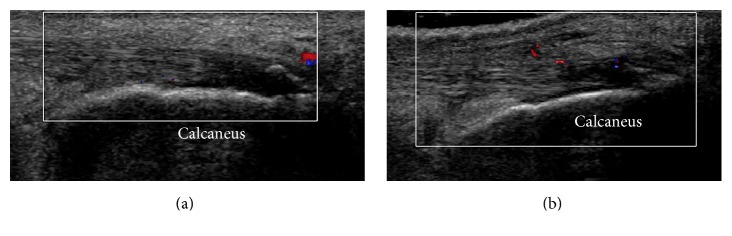
(a) Color Doppler shows no vessels in the insertion of the Achilles tendon before therapy. (b) There was neovascularization at the 4th week after therapy.

At the 4th and 12th week after therapy, there were significant improvements in VISA-A scores (*P* < 0.01). The mean diameters of calcific plaques decreased at follow-up, as shown by sonography (*P* < 0.05) (Figure 2). The percentage of patients with neovascularization increased to 90.5% at the 4th week and then decreased to 81.0% at the 12th week (Figure 3). The neovascularization scores increased at the 4th week (*P* < 0.01). However, the thickness and CSA of the Achilles tendon and the tendon structure score were unchanged (*P* > 0.05) (Table 1).

**Figure 4:**
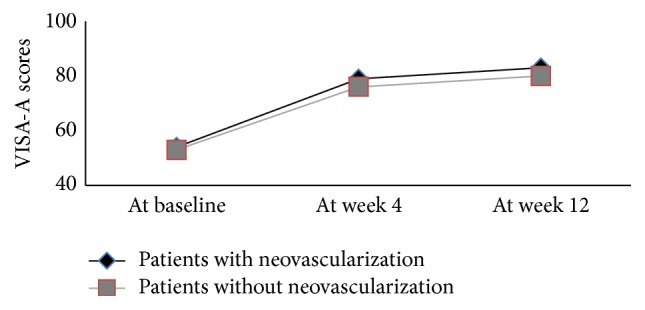
Changes in the VISA-A scores of patients with and without neovascularization at baseline, week 4, and week 12.

**Figure 5:**
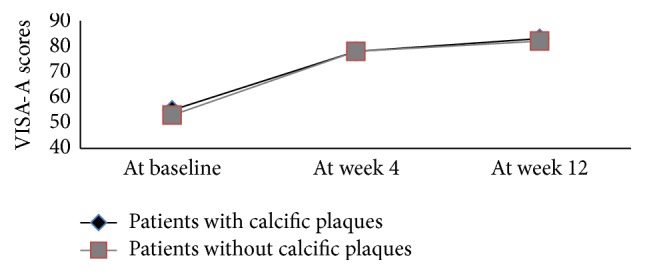
Changes in the VISA-A scores of patients with and without calcific plaques at baseline, week 4, and week 12.

The increased VISA-A scores at follow-up did not differ between patients with and without neovascularization or calcific plaques (Figures 4 and 5). The neovascularization scores or the size of calcific plaques at baseline was also not related to the increased VISA-A scores at follow-up. The thickness, CSA, and structure scores of the Achilles tendon at baseline were not associated with the changes in VISA-A scores at follow-up. However, the changes in calcific plaque size or neovascularization scores were related to the changes in VISA-A scores between pre- and posttherapy. There was a correlation between the decreased calcific plaque size and increased VISA-A scores at the 4th week (*r* = 0.71) and the 12th week (*r* = 0.68) (*P* < 0.01). The increased neovascularization scores at the 4th week were related to the increased VISA-A scores at the 4th week (*r* = 0.51) and the 12th week (*r* = 0.50) (*P* < 0.01). At the 12th week, the neovascularization scores decreased compared with the 4th week (*P* < 0.01). The difference in the neovascularization scores was not associated with the difference in VISA-A scores between week 12 and the baseline evaluation or between week 12 and week 4 (*P* > 0.05).

## 4. Discussion

There is no agreement on the best management for Achilles tendinopathy, with ESWT recently proposed as a viable treatment option [2–6]. In animal and in vivo experiments, some studies have reported that ESWT stimulated the ingrowth of neovascularization at the tendon-bone junction and promoted the inflammatory and catabolic processes [15, 16]. Others have demonstrated that ESWT significantly decreased the nonmyelinated sensory fibers and increased tenocyte proliferation, collagen synthesis, glycosaminoglycan (GAG) content, protein synthesis, and transforming growth factor- (TFG-) *β*1 [17–19].

In this study, the VISA-A scores of patients increased significantly after ESWT. Changes were also detected by US, such as increased neovascularization scores and decreased size of calcific plaques. In an animal study, Vetrano et al. [17] observed a significant increase in neovessels by biopsy at 4 weeks, and this increase in neovessels persisted until 12 weeks after ESWT. This result is comparable with that we detected at the 4th week. However, we observed a decrease in the neovascularization scores at the 12th week compared with the 4th week. The reduction of neovascularization did not influence the outcome. It is hypothesized that the number of blood vessels of the tendon was not reduced, but the percentage of slow blood flow, which cannot be detected with Color Doppler US, increased. The mechanical force of ESWT can disintegrate calcium deposits partially or completely, as applied to the treatment of chronic calcific tendinitis of the shoulder [20]. In our study, the size of calcific plaques also became smaller significantly at the 4th week. Ultrasonography revealed a trend of reduction in the thickness and CSA of Achilles tendon after therapy. However, the difference in the changes between pre- and posttherapy was not statistically significant, in accordance with the results of a study about ESWT for patellar tendinopathy [21]. The short duration of follow-up may be one reason for this result. The tendon structure was not changed significantly in this study. The hypoechoic areas in the tendons observed by US are caused by increased interfibrillar glycosaminoglycans and neovascularization, which can be induced by ESWT [22].

We attempted to evaluate the predictive value of US in ESWT in this study. Unfortunately, none of the categorical and continuous variables of US observed at baseline were associated with the changes in VISA-A scores at follow-up. Thus, we could not use US to predict the outcome of ESWT beforehand. However, at the 4th week, the increased neovascularization scores were related to the increased VISA-A scores. The neovascularization may improve the blood supply, leading to tissue regeneration in tendinopathy.

A limitation of this study is that the length of follow-up was short. In this study, we only used B-mode and Color Doppler US. Recently, sonoelastography was used to evaluate the mechanical properties of tendons [11]. We encourage further studies to assess outcome of therapy with sonoelastography.

## 5. Conclusion

Ultrasonography can reveal changes in the insertion of the Achilles tendon after ESWT, and the changes in calcific plaque size and neovascularization scores were related to the improvement of VISA-A scores at follow-up. However, the outcome of ESWT in IAT cannot be predicted by the variables observed by US.

## Figures and Tables

**Table 1:** Outcome after therapy.

Variables	Baseline	Week 4	*P*	Week 12	*P*
VISA-A scores	54.0 ± 8.0	78.3 ± 5.5	0.00	82.6 ± 5.6	0.00
Thickness of the Achilles tendon (mm)	3.8 ± 0.8	3.7 ± 0.9	NS	3.7 ± 0.9	NS
CSA of the Achilles tendon (mm^2^)	76.5 ± 17.4	76.7 ± 21.2	NS	73.1 ± 21.4	NS
Size of calcification (mm)	8.5 ± 8.4	7.3 ± 7.3	0.00	7.2 ± 7.2	0.00
Structure scores	2.1 ± 0.7	2.1 ± 0.7	NS	2.0 ± 0.7	NS
Neovascularization scores	1.3 ± 1.1	0.7 ± 0.9	0.00	0.6 ± 0.9	0.00

*Note*. Values are the mean ± SD; *P* value, comparison of data before and after treatment. *P* less than 0.05 was considered statistically significant. NS: not significant.
